# Intestinal microbiome-mediated resistance against vibriosis for *Cynoglossus semilaevis*

**DOI:** 10.1186/s40168-022-01346-4

**Published:** 2022-09-23

**Authors:** Qian Zhou, Xue Zhu, Yangzhen Li, Pengshuo Yang, Shengpeng Wang, Kang Ning, Songlin Chen

**Affiliations:** 1 Yellow Sea Fisheries Research Institute, Chinese Academy of Fishery Sciences/Key Laboratory for Sustainable Development of Marine Fisheries, Ministry of Agriculture; Shandong Key Laboratory for Marine Fishery Biotechnology and Genetic Breeding; Laboratory for Marine Fisheries Science and Food Production Processes, Pilot National Laboratory for Marine Science and Technology (Qingdao), Qingdao, 266071 Shandong China; 2 Key Laboratory of Molecular Biophysics of the Ministry of Education, Hubei Key Laboratory of Bioinformatics and Molecular-imaging, Center of AI Biology, Department of Bioinformatics and Systems Biology, College of Life Science and Technology, Huazhong University of Science and Technology, Wuhan, 430074 Hubei China; 3 Dezhou Key Laboratory for Applied Bile Acid Research, Shandong Longchang Animal Health Product Co., Ltd., Qihe, Shandong Lachance Co., Ltd., Jinan, 251100 Shandong China

## Abstract

**Background:**

Infectious diseases have caused huge economic loss and food security issues in fish aquaculture. Current management and breeding strategies heavily rely on the knowledge of regulative mechanisms underlying disease resistance. Though the intestinal microbial community was linked with disease infection, there is little knowledge about the roles of intestinal microbes in fish disease resistance. *Cynoglossus semilaevis* is an economically important and widely cultivated flatfish species in China. However, it suffers from outbreaks of vibriosis, which results in huge mortalities and economic loss.

**Results:**

Here, we used *C. semilaevis* as a research model to investigate the host-microbiome interactions in regulating vibriosis resistance. The resistance to vibriosis was reflected in intestinal microbiome on both taxonomic and functional levels. Such differences also influenced the host gene expressions in the resistant family. Moreover, the intestinal microbiome might control the host immunological homeostasis and inflammation to enhance vibriosis resistance through the microbe-intestine-immunity axis. For example, *Phaeobacter* regulated its *hdhA* gene and host *cyp27a1* gene up-expressed in bile acid biosynthesis pathways, but regulated its *trxA* gene and host *akt* gene down-expressed in proinflammatory cytokines biosynthesis pathways, to reduce inflammation and resist disease infection in the resistant family. Furthermore, the combination of intestinal microbes and host genes as biomarkers could accurately differentiate resistant family from susceptible family.

**Conclusion:**

Our study uncovered the regulatory patterns of the microbe-intestine-immunity axis that may contribute to vibriosis resistance in *C. semilaevis*. These findings could facilitate the disease control and selective breeding of superior germplasm with high disease resistance in fish aquaculture.

Video Abstract

**Supplementary Information:**

The online version contains supplementary material available at 10.1186/s40168-022-01346-4.

## Background

The world is currently facing increasing challenges to nutrition and food security issues [1, 2]. Aquaculture, which provides aquatic foods, plays an increasingly important role in the global food supply [3, 4]. As reported by the Food and Agriculture Organization, aquaculture production is projected to reach 109 million tons in 2030 [5], requiring a sustainable development of farming practices. However, infectious diseases occur frequently and are devastatingly affecting the aquaculture industry worldwide [6]. Vibriosis, caused by *Vibrio* species [7], is one of the most important threats in aquaculture, leading to huge mortalities and economic losses for a wide range of aquatic animals such as crustaceans, mollusks, and fishes [8, 9].

The intestinal microbiome is a pivotal and direct regulator of fish physiology, immunity, and health [10, 11]. Recently, the relevance of the microbiome to host has been identified as one of the most promising scientific breakthroughs that could have the greatest positive impact on food and agriculture [12]. With respect to the infectious diseases in fish aquaculture, understanding the divergence of the microbiomes between the resistant and non-resistant germplasms, and the interactions between the fish and microbiomes could provide important knowledge for disease control, probiotics development, genetic modification, and finally, boost the disease resistance and increase the production. Moreover, very few genetic markers are currently available to discriminate the disease resistance of the candidate fish for selective breeding. The investigation of fish intestinal microbiome and host-microbiome relationships may provide microbial and genetic biomarkers for discriminating the resistant germplasm, which can be applied in breeding practice.

The alterations of intestinal microbial community with disease infection status were reported in both model species like zebrafish [13] and economic species such as Atlantic salmon (*Salmo salar*) [14], grass carp (*Ctenopharyngodon idellus*) [15], and Chinese tongue sole (*Cynoglossus semilaevis*) [8]. Gaulke et al. demonstrated that the *Pseudocapillaria tomentosa* infection disrupts zebrafish intestinal microbiome composition, and the magnitude of microbiome disruption during infection varies with infection severity [13]. Gong et al. have found that the microbial community composition in *C. semilaevis* was significantly altered upon *Vibrio vulnificus* infection, and in the high-dose infection, the host could stimulate the innate immunity to inhibit *V. vulnificus* growth [16]. However, these shreds of evidence mainly focused on characterizing the intestinal microbial community composition and variation upon pathogenic infection using the 16S rRNA sequencing method, while the regulation patterns and underlying mechanism of the intestinal microbiome in resisting vibriosis in fish remain enigmatic.

On the other hand, fish resistance against pathogenic infections is a highly complex trait. Previous studies have demonstrated that the immune parameters, including the lysozyme activity, phagocytosis, and antibody level, against *Aeromonas hydrophila* infection, were different between resistant and susceptible common carp [17]. Several studies have pointed out the transcriptomic differences in response to different pathogenic infections. For example, the resistant Atlantic salmon showed a moderate and effective inflammatory response upon infectious pancreatic necrosis virus infection [18]. Resistant and susceptible animals showed distinct transcriptomic responses to CyHV-3 virus infections in the spleen of common carp [19]. Moreover, Han et al. detected that most differentially expressed genes (DEGs) in *C. semilaevis* were annotated to the signal transduction pathways after *Shewanella algae* infection [20]. Current knowledge on disease resistance in fish was mainly focused on physiological and transcriptomic responses after infections, while few studies have investigated the function of the intestinal microbes against disease infection.

Chinese tongues sole (*C. semilaevis*) is an important and widely cultivated economic flatfish species with delicious taste and superior nutritive value, which is recorded as one of the nine varieties in the national marine fish industry technology system of China (https://www.cafs.ac.cn/info/1024/38584.htm). However, this species is suffering from the outbreaks of vibriosis caused by *Vibrio harveyi* [21, 22], which has resulted in high mortalities (> 70%). In our previous work, we conducted successive selective breeding for five generations with artificial infection method from 2005 to 2016 and obtained vibriosis-resistant and susceptible *C. semilaevis* families [22]. We have also presented the evolutionary divergence and genetic architecture underlying the phenotypic improvement in vibriosis resistance in *C. semilaevis* [21, 22]. Thus, these materials provide a unique opportunity to investigate the divergence of microbiomes between the resistant and susceptible families and the associations of host and intestinal microbes in regulating vibriosis resistance in fish.

**Fig. 1:**
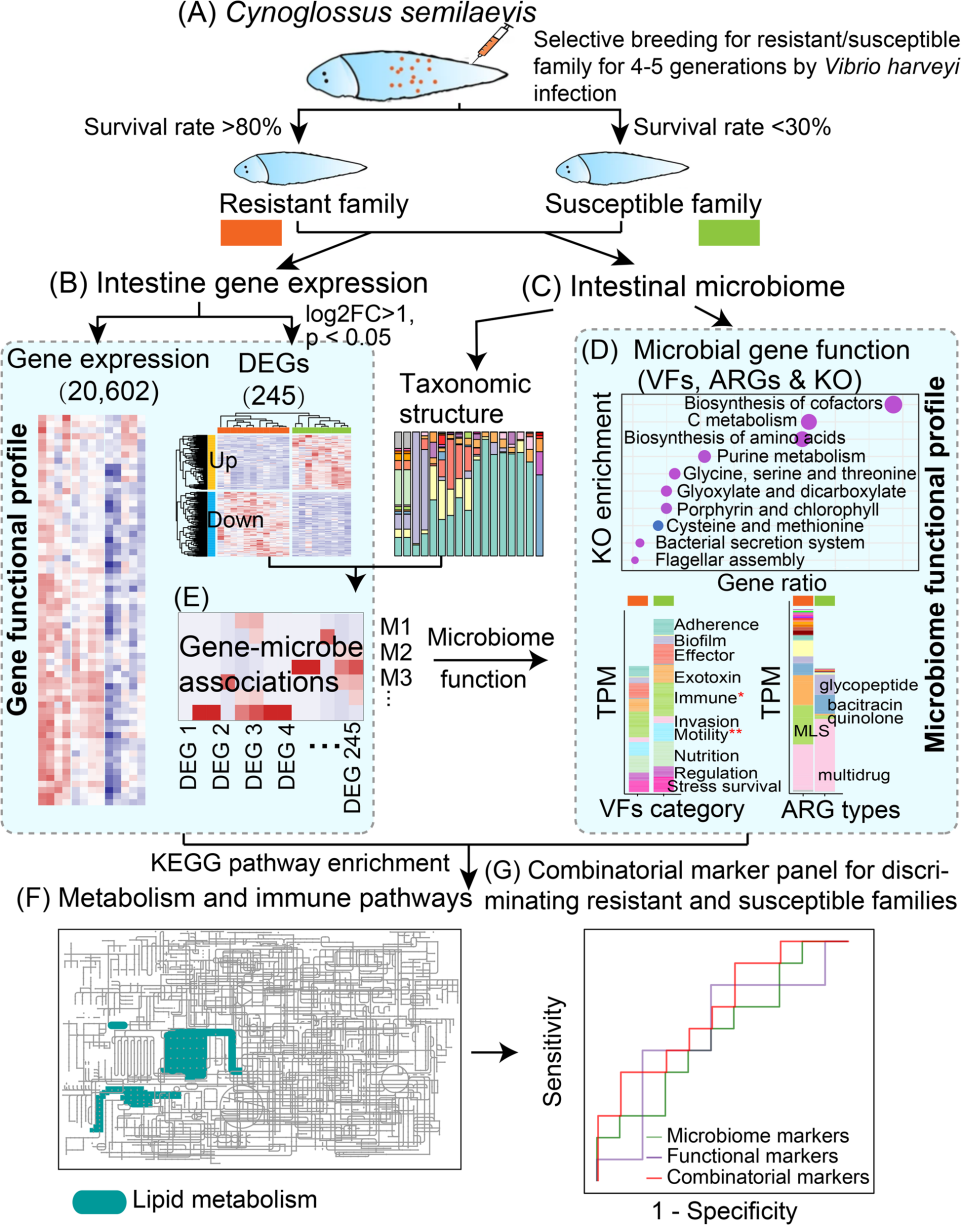
Overview of the workflow by integrating multi-omics including host genes, intestinal microbes, and intestinal microbial genes. **A** Experimental design for selective breeding of the *V. harveyi* resistant and susceptible families in *C. semilaevis*. The selective breeding was performed for successive 4–5 generations over 10 years. The family having a survival rate > 80% and < 30% was considered as a *V. harveyi* resistant family and susceptible family, respectively. **B** The transcriptomic analysis of *C. semilaevis* genes in the resistant and susceptible families. **C** Profiling of the intestinal microbial communities for the resistant and susceptible families. **D** The functional analysis includes virulent factors (VFs), antibiotic-resistant genes (ARGs), and the KEGG pathway for intestinal microbial genes. **E** The association analysis of the host’s differentially expressed genes (DEGs) and intestinal microbes. The DEGs with |log_2_(FoldChange)| > 1 and the adjusted *p* < 0.05, as well as the microbes with relative abundance > 0.1% and coverage of > 10% samples were selected for this association analysis. **F** Metabolism pathway enrichment analysis by integrating host genes, intestinal microbes, and intestinal microbial genes. **G** The prediction power of intestinal microbial markers, host functional markers, and their combinatorial markers in discriminating the resistant family from the susceptible family for *C. semilaevis*

Here, we collected intestinal tissue samples, as well as their intestinal content from both resistant and susceptible families of *C. semilaevis* (Fig. 1A) and conducted a multi-omics study to address the following issues: (1) How are the host intestinal gene expressions (Fig. 1B), as well as the intestinal microbiome taxonomic and functional profiles (Fig. 1C, D), different between the resistant and susceptible families? (2) How do the intestinal microbes influence the host genes to resist vibriosis (Fig. 1E, F)? 3) Are there any interpretable microbial and functional markers that could accurately differentiate resistant and susceptible families (Fig. 1G)? To the best of our knowledge, our study represents one of the first studies revealing the patterns of the interactions of host genes and intestinal microbes in regulating infectious disease resistance in fish. The microbial and host functional features we identified could effectively facilitate disease control and further selective breeding practice for superior germplasms of *C. semilaevis*.

## Methods

### Sample collection

The selective breeding of the *C. semilaevis* resistant and susceptible families, and the evaluation of their resistance against *V. harveyi* were performed as previously described [22]. Briefly, the genetic sex of parental fish was identified by a sex-specific AFLP marker [23]. Each family was cultured in separate tanks until the juvenile stage, then tagged with visible implant elastomers and reared in several larger common tanks under a flow-through system. When fish reached an average size of 10–12 cm, challenge tests were performed to examine the resistance to *V. harveyi* infection [22]. Families having a survival rate > 80% and < 30% were considered as resistant and susceptible families, respectively (Fig. 1A). After a successive selection for five generations, we collected 11 and 9 intestinal tissue samples, as well as their intestinal content from the resistant and susceptible families, respectively (Fig. 1 and Supplementary Table 1). The body weight (BW) and length (BL) were measured for all collected fish (Supplementary Table 1). All of the samples were stored at −80℃ before sequencing.

### Transcriptomic analysis of intestinal tissues

The total RNA was extracted from intestinal tissues using Trizol according to the manufacturer’s instructions. Pair-ended (PE) RNA-seq libraries were constructed using the Truseq mRNA-stranded RNA-Seq Library Prep Kit (Illumina, USA). The sequencing of the libraries with a read length of 2×150 bp and an insert size of 380 bp was performed on an Illumina HiSeq X Ten sequencing platform. The quality of the raw sequencing data was assessed and filtered with RNA-QC-Chain [24], removing the adaptors, contaminations, and low-quality reads. The filtered reads were aligned to the reference genome of the *C. semilaevis* (NCBI Accession No. GCA_000523025.1) [25] using HISAT2 (v2.1.0) [26]. The expression level of genes was estimated using fragments per kilobase per million mapped reads (FPKM) by StringTie (v1.3.6) [27]. We used DESeq2 (v.3.4) [28] to detect the differentially expressed genes (DEGs), which were defined as genes with |log_2_(FoldChange)| > 1 and adjusted *p* < 0.05 between resistant and susceptible families. The Benjamini-Hochberg (BH) method-based false discovery rate (FDR) multiple test correction was applied to adjust the *p* value. Kyoto Encyclopedia of Genes and Genomes (KEGG) enrichment analyses were performed for these nonredundancy proteins using KOBAS (v3.0.3) [29], and the KEGG pathways with adjusted *p* < 0.05 were considered as enriched terms.

### Metagenomic analysis of intestinal microbiome

The total DNA from the intestinal content samples was extracted using QIAamp DNA Stool Mini Kit (Qiagen, Germany) following the manufacturer’s instructions. After the quality check, DNA was used to construct the PE libraries using NEBNext Ultr DNA Library Prep Kit for Illumina (NEB, USA). A total of 17 samples (Supplementary Table 1) were successfully sequenced on Illumina HiSeq X Ten platform, generating 128.04 Gb raw reads. The raw sequencing data were evaluated using FastQC (v0.11.6) [30] and then trimmed by Trimmomatic (v0.38) [31] to eliminate reads less than 100 bp, adapters, leading or trailing bases with Phred base quality (BQ) scores of < 20, and fragments of every five bases with an average BQ score of < 25. After preliminary quality control, we obtained approximately 121.90 Gb and 4.15 billion high-quality PE clean reads.

To obtain the taxonomical composition of the intestinal microbiome for each sample, the high-quality reads (94.93% of the raw reads) were annotated by MetaPhlAn2 (v2.6.0) with default settings [32]. If a taxon was unclassified at the genus level but classified at the family level, the prefix “uc_” (unclassified_) was added in its taxon name, such as *uc_Rhodobacterales*. Shannon index-based alpha diversity was calculated using the R (v4.0.3) diversity() function. Wilcoxon test was applied to detect the variations in the intestinal microbiome between the resistant and susceptible families. Microbes with a relative abundance (RA) of ≥ 1.00% and coverage of > 10% samples were selected for co-occurrence network analysis. Spearman rank correlation was used to calculate the microbial correlations for the resistant and susceptible families. Only Spearman correlations of ≥ 0.65 or ≤ 0.65 with *p* < 0.05 were considered as strong correlations and were visualized in Cytoscape (v3.8.1) [33].

### Intestinal microbial function analysis

The high-quality reads for each sample were also assembled into contigs using MEGAHIT (v1.1.2) [34] with “-min-contig-len 1000”. The assemblies were evaluated using QUAST (v5.0.2) [35]. All resulting assemblies were subsequently clustered into genome bins individually using MetaWRAP (v1.2.2) [36] with default settings. In total, 49 individual genome bins were assembled across all samples. Each bin was assigned to the highest-scoring taxonomy by Kraken2 (v2.0.8-beta) [37]. Open reading frames were predicted using metaProdigal (v2.6.3) [38] with “-p meta”. The predicted genes and proteins were then dereplicated and clustered into the most similar cluster at 95% identity and 90% sequence coverage using CD-HIT (v4.8.1) [39]. The abundance (measured by fragments per million (TPM)) of non-redundant genes was calculated by Salmon (v1.3.0) [40]. ComBat (R package “sva”; v3.38.0) [41] was used to correct the effect of sequencing depth on calculating the abundance of intestinal microbial functional genes. The non-redundant proteins were assigned to the KEGG database using KofamKOALA (v1.3.0) [42] for KO annotation. We also aligned the non-redundant proteins into HMD-ARG database (v5) [43] using BLASTp (v2.7.1+) with “-e 1e-5” for antibiotics resistance gene (ARG) detection. To detect the virulent factor (VF) of each microbe, the non-redundant proteins were aligned against the VF database (VFDB: VFDB_setB_pro.fas) [44] using diamond (v0.9.21.122) [45]. The VF annotation of a gene was using its best hit alignment with the identity greater than 90% and an *e* value below 1e−5.

### Integrated analysis of the association between host genes and intestinal microbiome

We used Spearman rank correlation analysis to investigate the link between the host intestinal DEGs (|log2(FoldChange)| > 1 and adjusted *p* < 0.05) and intestinal microbes (RA ≥ 0.1% and coverage of > 10% samples). We also used the cor.test() function in R (v4.0.5) to compute the Spearman rank correlations and *p* values with the two-sided alternative hypothesis to detect their strong associations (adjusted *p* < 0.05).

### Prediction model for discriminating the resistant and susceptible families

To evaluate the potential role of the intestinal microbiome and functional genes in discriminating the resistant and susceptible families, we used the microbial community composition and the microbe-associated host DEGs, and their combinations to develop the random forest models using the R package “randomForest ”[46]. To avoid overfitting, we used 70% samples for training and 30% samples for testing. According to MeanDecreaseAccuracy and MeanDecreaseGini of the random forest model, the markers with outstanding performance in individual prediction were selected for a combinatorial marker panel. The links between the combinatorial marker panel and the phenotypic characters (represented by BW and TL) were also investigated, and only the Spearman rank correlations with adjusted *p* < 0.05 were considered as strong correlations.

## Results and discussion

### Transcriptomic differences between resistant and susceptible families

**Fig. 2:**
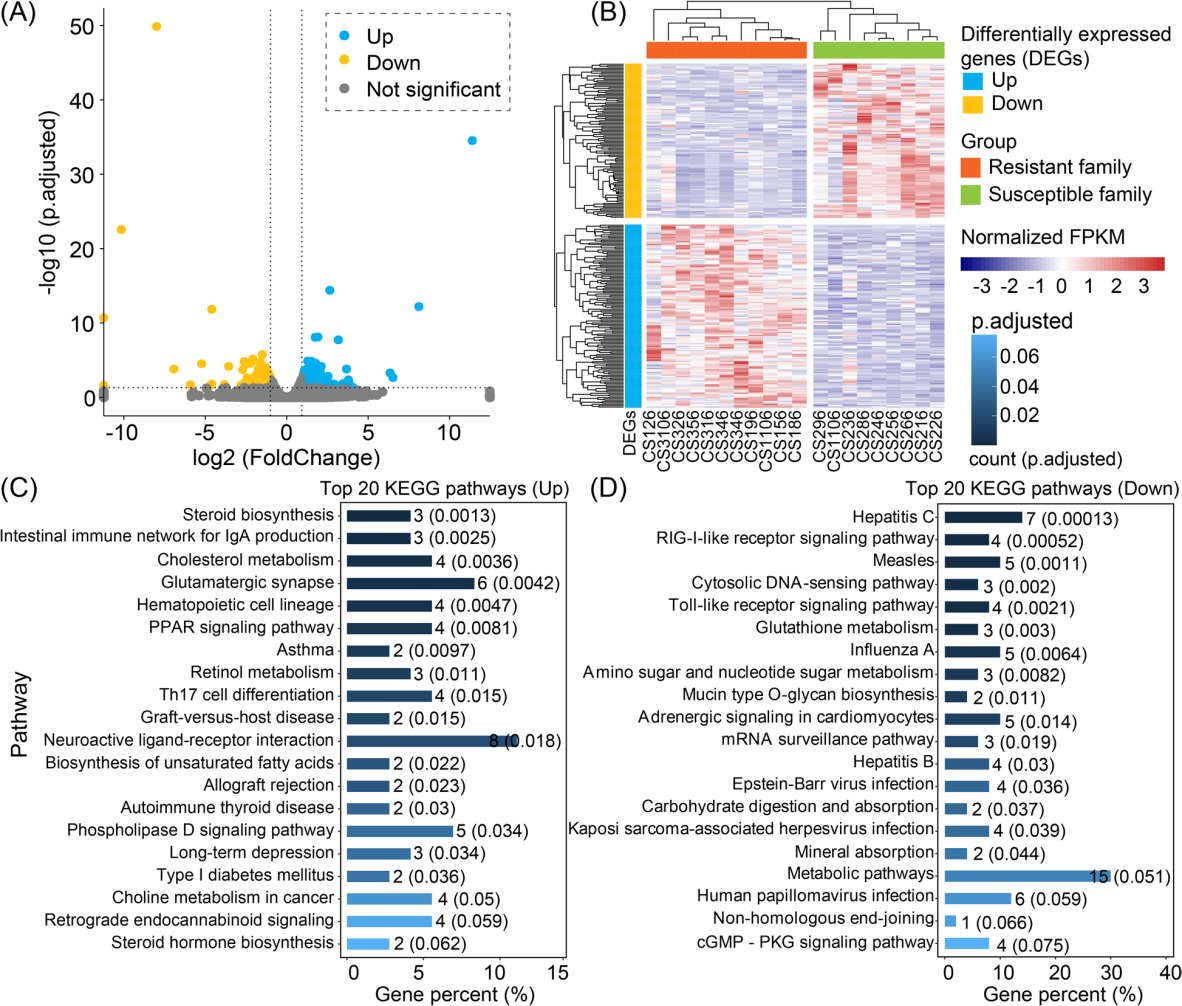
Differentially expressed genes (DEGs) distribution and their associated functions in *C. semilaevis*. **A** Volcano plot for DEGs between resistant and susceptible families*.* The DEGs with |log_2_(FoldChange)| >1 and adjusted *p* < 0.05 were colored in blue (up-expressed DEGs in the resistant family) and yellow (down-expressed DEGs in the resistant family), respectively*.* **B** The DEG composition between resistant family (orange) and susceptible family (green). **C** Top 20 KEGG enrichment pathways (adjusted *p* < 0.05) of the up-expressed genes in the resistant family. **D** Top 20 KEGG enrichment pathways (adjusted *p* < 0.05) of the down-expressed genes in the resistant family. For each bar in **C** and **D**, the numbers indicate the number (percent) of genes in the pathway, while the numbers in parenthesis represent the adjusted *p* values

By comparing the gene expression profiles in the host intestinal tissues, we identified 245 DEGs (|log_2_(FoldChange)| >1 and adjusted *p* < 0.05) between the resistant and susceptible families of *C. semilaevis* (Fig. 2A). Among them, 133 and 112 genes were up- and down-expressed in the resistant family, respectively (Fig. 2A and B). We observed that these up- and down-expressed genes were enriched in different KEGG functional categories (Fig. 2C, D). The up-expressed genes in the resistant family were significantly enriched in lipid metabolism pathways, such as steroid and steroid hormone biosynthesis, cholesterol metabolism, and biosynthesis of unsaturated fatty acids (UFAs) (*p* < 0.05; Fig. 2C), in which the involved DEGs encode the sterol O-acyltransferase (SOAT), cholestanetriol 26-monooxygenase (CYP27A1), apolipoprotein A-IV (APOA4), and methylsterol monooxygenase (MESO1) (Supplementary Table 2). Another significantly enriched category for up-expressed genes was immune system, such as intestinal immune network for IgA production and hematopoietic cell lineage (*p* < 0.05; Fig. 2C), in which the involved DEGs encode the MHC class II antigen (MHC2) and C-X-C chemokine receptor type 4 (CXCR4) (*p* < 0.05; Fig. 2C and Supplementary Table 2). In terms of the down-expressed genes in the resistant family, clear enrichments were observed in three immune-related signaling transduction pathways, including RIG-I-like receptor signaling pathway, cytosolic DNA-sensing pathway, and Toll-like receptor signaling pathway (*p* < 0.05) (Fig. 2D), in which the DEGs encode the interferon regulatory factor (IRF) 3, IRF7, signal transducer and activator of transcription 1 (STAT1), and RAC serine/threonine-protein kinase (AKT) (Supplementary Table 2). Taken together, these results indicated that the resistant and susceptible families have significantly different host gene expression patterns with distinct functional implications.

### Differences in intestinal microbial community composition between resistant and susceptible families

**Fig. 3:**
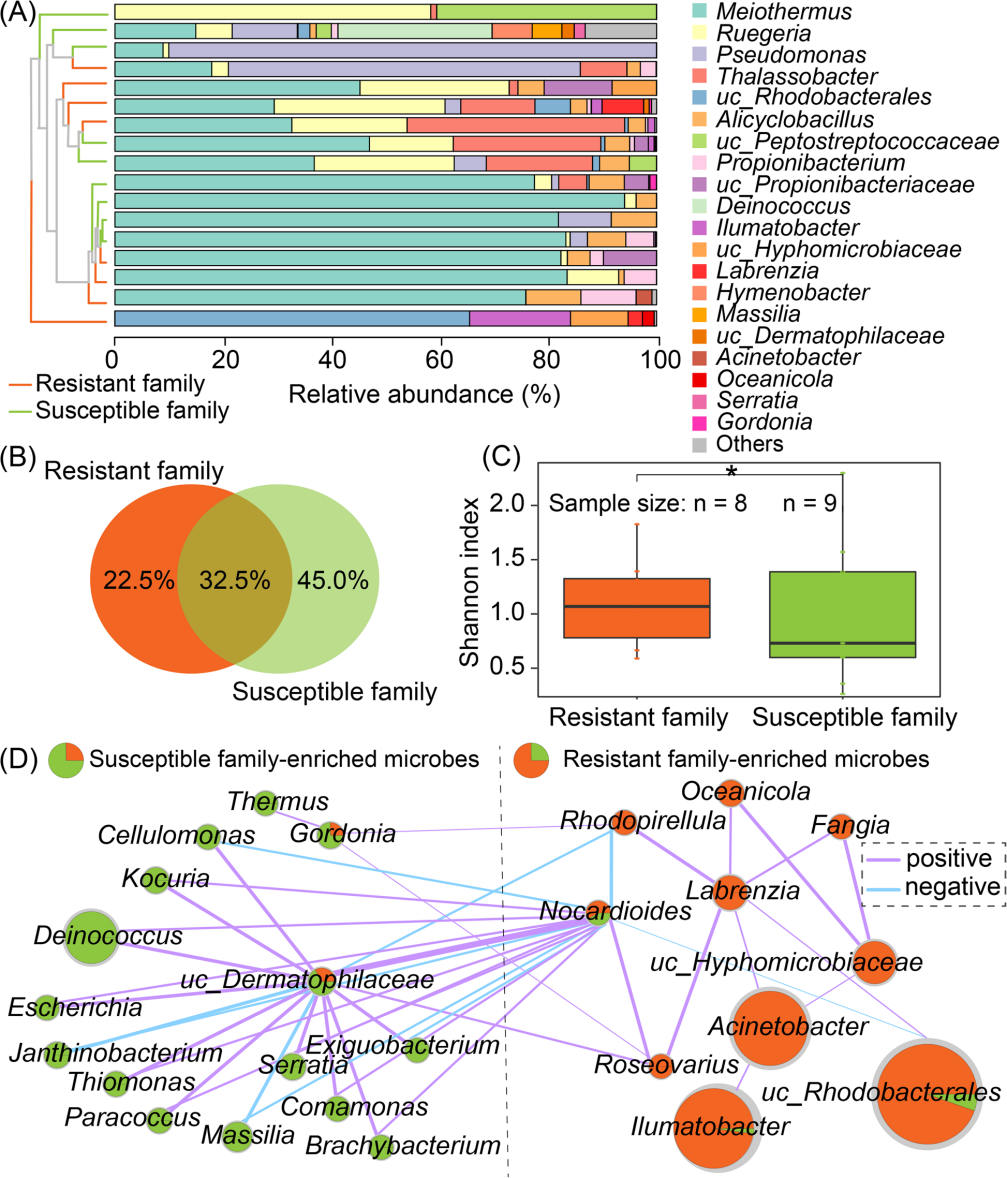
*C. semilaevis* intestinal microbial community composition, diversity, and co-occurrence network. **A** Microbial community compositions in resistant and susceptible families. Unweighted paired-group method with arithmetic means (UPGMA)-based hierarchical clustering (Bray-Curtis distance) was used to cluster the samples according to the microbial community structure. **B** Venn plot illustrated the shared microbes and specific microbes of resistant and susceptible families. **C** Shannon index-based microbial diversity. Wilcoxon test was used to detect the variation between resistant and susceptible families. “*”*p* < 0.1. **D** Microbial co-occurrence network of resistant and susceptible families. The co-occurrence network is built based on the microbes with relative abundance ≥ 1.00% and coverage of > 10% samples using the Jaccard coefficient as the distance measurement. Only the Jaccard coefficient with an absolute value ≥ 0.6 and *p* < 0.05 was considered as significant correlations and was visualized in the network. Purple edges indicate positive associations, whereas blue edges reflect negative associations. The size of the nodes indicates the relative abundance of the microbes. The nodes are divided into two portions according to the relative abundance of microbes between resistant and susceptible families (orange portion: resistant family-enriched microbes; green portion: susceptible family-enriched microbes). All of the microbes shown in this figure are based on microbial taxonomical information at the genus level

The intestinal microbial communities between resistant and susceptible families were also distinct. At the phylum level, Actinobacteria (relative abundance (RA): 8.60 vs. 2.45%) was more abundant in the resistant family, while Firmicutes (RA: 3.61 vs. 9.50%) and Bacteroidetes (RA: 0 vs. 0.82%) were detected with higher abundances in the susceptible family. At the genus level, 13 genera (32.5% out of all the identified genera) were shared by the resistant and susceptible families, and nine (22.5%) and 18 genera (45.0%) were specific in resistant and susceptible families, respectively (Fig. 3A, B). While *uc_Rhodobacterales* (RA: 9.08 vs. 4.55%), *Propionibacterium* (RA: 2.85 vs. 1.77%), *uc_Propionibacteriaceae* (RA: 2.81 vs. 1.75%), *uc_Hyphomicrobiaceae* (RA: 2.35 vs. 1.11%), and *Phaeobacter* (RA: 0.01 vs. 0.08%) were more abundant in the resistant family, *uc_Peptostreptococcaceae* (RA: 0 vs. 5.38%) and *Deinococcus* (RA: 0 vs. 3.16%) were detected with a higher prevalence in the susceptible family. Besides, 22.5% of genera were identified from Actinobacteria, while the bacteria from Actinobacteria were reported as good probiotics for resisting vibriosis [16], which might help *C. semilaevis* adapt to the vibriosis environment. Moreover, the resistant-enriched microbes *Propionibacterium* and *Phaeobacter* were reported as probiotics to establish beneficial microbes and promote the development of the intestinal mucosa and the immune system against pathogen infection [47–50], while the susceptible-enriched microbe *Alicyclobacillus* could promote inflammation by eliciting an interleukin 6 (IL 6) response [51]. These findings suggested the family-specific intestinal microbiome may be related to host immunity to resist disease infection.

The obvious differences between resistant and susceptible families could also be found in alpha-diversity (Wilcoxon test, *p* < 0.1; Fig. 3C) and the co-occurrence network (Fig. 3D). The microbes *uc_Dermatophilaceae* and *Nocardioides* from Actinobacteria were positively correlated with most of the other microbes, thus might act as key microbes in the network (Fig. 3D). Additionally, we also found most of the members in the network were from Actinobacteria, which emphasized that the intestinal microbial communities may recruit the probiotic microbes from Actinobacteria [16] and form their unique microbial community to resist disease infection.

### Intestinal microbial gene differences between resistant and susceptible families

**Fig. 4:**
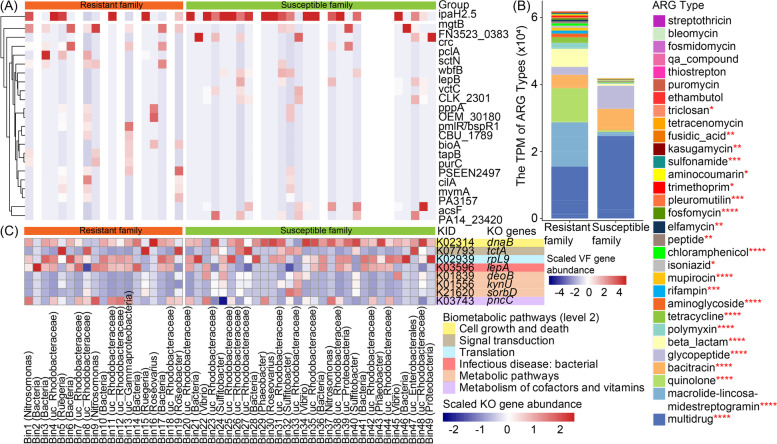
The potential functional roles in the intestinal microbiome between resistant and susceptible families in *C. semilaevis*. **A** The distribution of virulent factor (VF) genes in resistant and susceptible families. **B** The abundance (fragments per million; TPM) of antibiotic resistance gene (ARG) type in resistant and susceptible families. Wilcoxon test was used the detected the significant variations in the ARG types between the resistant and susceptible families in *C. semilaevis*. The ARGs labeled with asterisks were significantly varied in abundance between the resistant and susceptible families. “**”*p* < 0.05; “****”*p* < 0.001. **C** The differentially expressed microbiome genes between resistant and susceptible families. Gene abundances covered more than 20% of bins were assessed for significant elevation or depletion in bio-metabolic pathways between resistant and susceptible families. Only the relative abundance of KO genes with significant differences (Wilcoxon test, *p* < 0.05) between two *C. semilaevis* families were shown in the heatmap

To further understand the potential role of host-microbiome interaction against *V. harveyi* infection, we next investigated the differences in microbial genes between resistant and susceptible families. We found that 24 VF genes in the intestinal microbes were significantly different between resistant and susceptible families (Fig. 4A). Among them, *ipaH2.5* was significantly enriched in the susceptible family (Wilcoxon test, *p* < 0.05, Fig. 4A). Previous studies have reported that *ipaH2.5* could promote the degradation of Heme-oxidized IRP2 ubiquitin ligase-1-interacting protein, which would induce the irreversible inactivation of NF-κB, TNF, and IL-1β [52]. NF-κB is widely reported to initiate inflammatory responses and regulate the expression of proinflammatory cytokines (such as TNF, IL-6, and IL-8) [53]. Thus, the high abundance of *ipaH2.5* might indirectly reduce the level of inflammatory cytokines in the susceptible family.

We also detected 31 ARG types and 444 ARG subtypes in both resistant and susceptible families (Fig. 4B). An average of 76.08 multi-drug antibiotic resistance genes (ARGs) per sample were detected, which was the most prevalent type, followed by macrolide-lincosamide-streptogramin (MLS) (39.84), beta-lactam (26.04), bacitracin (25.47), quinolone (16.67), and glycopeptide (16.16). Though the intestinal microbes in the resistant families contained higher levels of ARGs than that of the susceptible family, there was no significant difference in ARG diversity (Fig. 4B and Supplementary Figure 1). We observed that 48.49% of ARGs could exert resistance to the changing environment mainly through antibiotic efflux, then 34.66% and 11.14% of them were through antibiotic target alteration and antibiotic inactivation, respectively.

Apart from the VF genes and ARGs, the microbial genes mapped to the KEGG pathway showed the family-specific pattern in *C. semilaevis* (Supplementary Figure 2). Besides, we detected 8 genes (covering > 20% of bins) that have significantly different abundances between the resistant and susceptible families (Wilcoxon test, *p* < 0.05; Fig. 4C, and Supplementary Table 3). Among them, *dnaB*, putative tricarboxylic transport membrane protein (*tctA*), large ribosomal protein L9 (*rpL9*), *deoB*, kynureninase (*kynU*), and *sorbD* were detected with a higher abundance in the susceptible family, while *lepA* and *pncC* were enriched in the resistant family (Wilcoxon test, *p* < 0.05; Fig. 4C and Supplementary Table 3). Notably, *kynU* is reported to contribute to inflammation [54]; thus, its high abundance might reflect a higher inflammation level in the susceptible family. Moreover, *lepA* involved in bacteria-mediated infectious disease (Supplementary Table 3) could identify the defective translocation of ribosomes and induce a back-translocation for correcting protein biosynthesis [55], which is important for bacterial growth and functional protein biosynthesis [56]. The high abundance of *lepA* in the resistant family enables the high-quality biosynthesis of bacterial functional proteins, which may indirectly facilitate the microbiome to resist *V. harveyi* infection. In summary, these intestinal microbiome genes may play roles in regulating inflammation and the biosynthesis of proteins, which contributes to the differential resistant capacity between the resistant and susceptible families.

### Associations between host DEGs and intestinal microbes

**Fig. 5:**
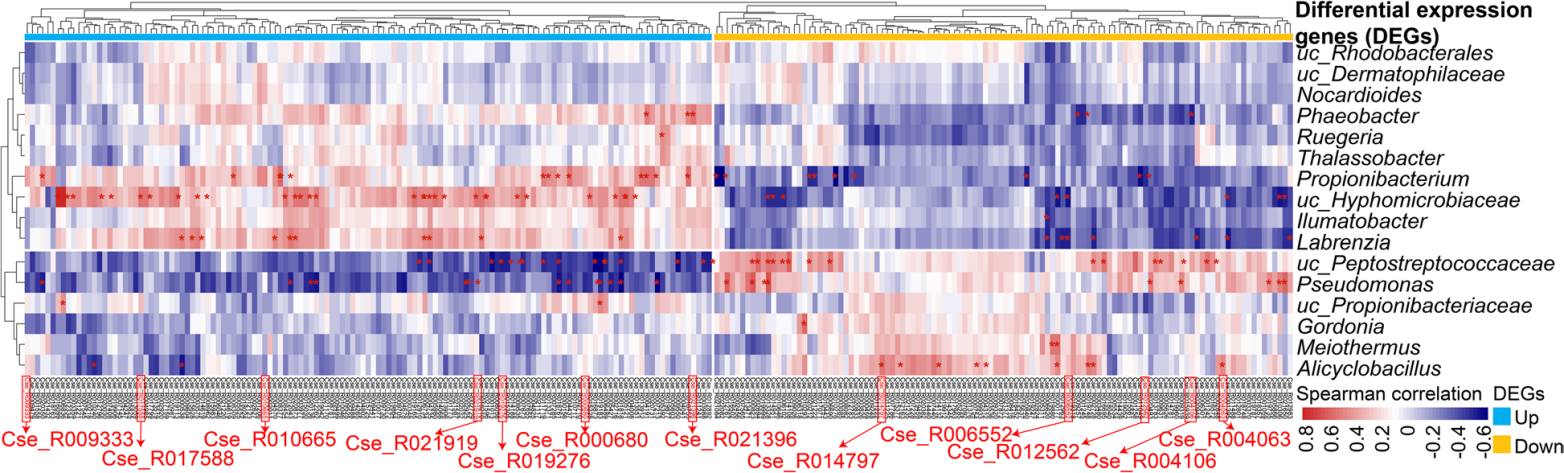
The host intestine DEGs have strong family-specific associations with the intestinal microbiome in *C. semilaevis.* We selected the genus with relative abundance ≥ 0.1% and coverage > 10% samples, and the DEGs with |log2(FoldChange)| > 1.00 and adjusted *p* < 0.05 for correlation analysis. The horizontal bar in yellow and blue represents the gene with down- and up-expression in the resistance family, respectively*.* Only the Spearman rank correlations with *p* < 0.05 were considered as strong associations and were marked with “*” in the heatmap (*p* < 0.05). The red and blue cells in the heatmap indicate that the genes were positive and negatively correlated with microbes, respectively. The red genes indicated those enriched in the lipid metabolism and immune signaling transduction pathways

In order to investigate the correlations between host genes and intestinal microbiome, and their potential roles in vibriosis resistance, we explored the associations between 245 DEGs (|log2(FoldChange)| > 1.00, adjusted *p* < 0.05) and 16 microbes (RA ≥ 0.1% and coverage > 10% samples). We found resistant and susceptible family-specific host-microbiome associations, among which, 116 associations were considered as strong ones (Spearman rank correlation ≥ 0.5 or ≤ −0.5, hypothesis test *p* < 0.05; labeled with “*” in Fig. 5). Interestingly, most microbes associated with the up- and down-expressed DEGs in the resistant family were also distinct (Fig. 5). That is, ten microbes (*uc_Rhodobacterales*, *uc_Dermatophilaceae*, *Nocardioides*, *Phaeobacter*, *Ruegeria*, *Thalassobacter*, *Propionibacterium*, *uc_Hyphomicrobiaceae*, *Ilumatobacter*, and *Labrenzia*) had positive associations with most up-expressed genes, but were negatively associated with the down-expressed genes in the resistant family (Fig. 5). On the contrary, six microbes (*uc_Peptostreptococcaceae*, *Pseudomonas*, *uc_Propionibacteriaceae*, *Gordonia*, *Meiothermus*, and *Alicyclobacillus*) were positively associated with the down-expressed genes, but were negatively associated with the up-expressed genes in the resistant family (Fig. 5). These results implied that these intestinal microbes might interact with the DEGs to regulate vibriosis resistance.

### Regulative network of microbe-intestine-immunity axis underlying vibriosis resistance

**Fig. 6:**
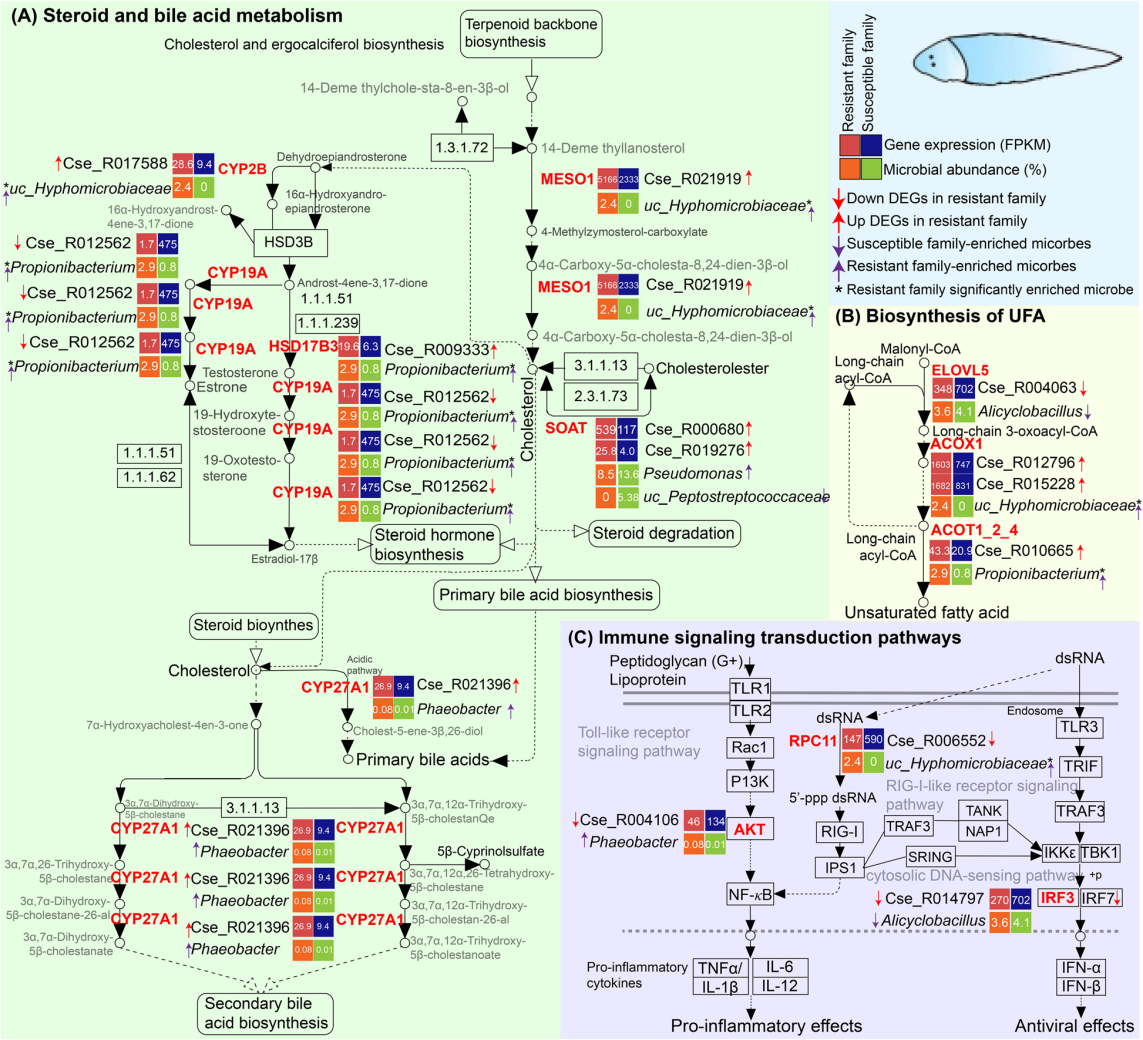
Proposed regulatory networks involved in anti-infection associated metabolism pathway in *C*. *semilaevis*. **A** Steroid, steroid hormone, and bile acid (BA) biosynthesis metabolism. **B** Biosynthesis of unsaturated fatty acids (UFAs). **C** Immune signaling transduction pathways. The enriched pathway of the DEGs (|log_2_(FoldChange)| > 1 and adjusted *p* < 0.05) that have significant associations (Spearman rank correlations ≥ 0.5 or ≤ −0.5, hypothesis test, *p* < 0.05) with microbes (relative abundance ≥ 0.1% and coverage > 10% samples) were selected. Differences in host gene expression and microbe abundances in two families are shown along the pathway: the red bold words were the KOs that were involved in the anti-infection associated metabolism pathways. The upward and downward red arrows indicate the DEGs were up-expressed and down-expressed in the resistant family, respectively. While the upward and downward purple arrows indicate the microbes were more abundant in the resistant (orange box) and susceptible families (green box), respectively. The red and blue boxes in the top right sub-figure represent the abundance (TPM) of genes in the resistant and susceptible families, respectively. While the orange and green boxes in the top right sub-figure represent the relative abundance of intestinal microbes in the resistant and susceptible families, respectively. “*” means the microbes were significantly differentiated (*p* < 0.05) in relative abundance between the resistant and susceptible families of *C. semilaevis*

We proposed the microbe-intestine-immunity axis for a deeper understanding of the regulative network of the host-microbiome interaction against vibriosis. Our analyses have already indicated that most host DEGs, family-specific intestinal microbes, and microbial function were related to immune response to resist pathogenic infection. Here, we have employed KEGG enrichment analysis, and the results revealed that most of the DEGs were enriched in the lipid metabolism, including steroid, steroid hormone, and bile acid (BA) metabolism (Fig. 6A), biosynthesis of UFAs (Fig. 6B), and several immune signaling transduction pathways, including the Toll-like signaling pathway, RIG-I-like receptor signaling pathway, and cytosolic DNA-sensing pathway (*p* < 0.05; Fig. 6C). And these DEGs have strong correlations with intestinal microbes (Figs. 5 and 6). These results indicated that the intestinal microbiome may regulate these pathways to resist *V. harveyi* infection.

### Steroid and bile acid metabolism

In the steroid and BA metabolism pathways, five host genes, including *meso1*, *soat*, *cyp2b*, *hsd17b3*, and *cyp27a1*, were significantly up-expressed, while *cyp19a* was down-expressed in the resistant family (*p* < 0.05, Fig. 6A). MESO1 catalyzes the three-step monooxygenation required for cholesterol biosynthesis, while SOAT is a bidirectional transformation of cholesterol and cholesterolester (Fig. 6A). It was reported that during the pathogen infection, the reverse cholesterol transport was inhibited [57], while the cholesterol accumulation, especially in the immune-associated cell, could induce and aggravate the inflammatory responses [58]. Another study has presented that during *A. hydrophila* infection in the grass carp, the gene expression of pro-inflammatory cytokines (i.e., IL6, IL8, and TNF-α) varied with the graded levels of cholesterol [59]. Furthermore, we identified the expression of *meso1* and *soat* genes were associated with the improved abundance of microbes *uc_Hyphomicrobiaceae* and *uc_Peptostreptococcaceae*, respectively (Fig. 6A), indicating that these bacteria may modulate the host cholesterol metabolism by regulating the *meso1* and *soat* expressions. These interactions may enable a proper level of cholesterol in the plasma [60, 61], which may support a high resistance against *V. harveyi* infection. In addition, in the steroid hormone biosynthesis pathway, the host intestinal DEGs *cyp2b*, *cyp19a*, and *hsd17b3* were associated with the abundance variations in *uc_Hyphomicrobiaceae* and *Propionibacterium* (Fig. 6A). Previous studies have revealed that the activation of steroid hormone signaling is essential for immune responses and survival after pathogenic challenge in *Drosophila* [62]. Therefore, these results indicated that the host-microbiome associations may play a role in steroid hormone regulation, which might impact host immunity to resist vibriosis.

Furthermore, we also detected that the intestinal microbes were associated with the genes involved in BA metabolism. BAs are synthesized from cholesterol in the liver and then released into the intestine [61]. Most of the BAs were re-absorbed through enterohepatic circulation, and they are critical in regulating lipid and glucose absorption and homeostasis [63]. Approximately, 5% of BAs are transformed into secondary BAs by intestinal microbiomes [64, 65], which could reduce intestinal inflammation. The *cyp27a1* gene is an enzyme that transforms the cholesterol into primary BA and/or a series of cholesterol derivatives, and these cholesterol derivatives finally synthesize the secondary BA (Fig. 6A). The up-expression of *cyp27a1* indicated an enhanced BA biosynthesis capacity in the resistant family (Fig. 6A). It was reported the BAs undergo bacteria-mediated transformations to generate bioactive immune signaling molecules to regulate host and intestinal immunological homeostasis [66]. Additionally, the host-microbiome biliary network may control both the innate and adaptive immune responses via resulting BA metabolites [59]. Interestingly, we also observed that the relative abundance of *Phaeobacter*, which was more abundant in the resistant family, was associated with the expression of *cyp27a1* (Spearman correlation = 0.55, *p* < 0.05; Fig. 6A). *Phaeobacter* was reported as a probiotic bacterium that can improve the disease resistance against vibriosis in aquaculture [49, 50]. Our metagenomic analysis also revealed that *Phaeobacter* could regulate the expression of its *hdhA* gene to influence the secondary BA metabolism (Supplementary Figure 3 and Supplementary Table 4), which may enhance the host vibriosis resistance. The multi-directional relationship between the intestine, along with its microbiota, and the BA-induced host immunity may benefit disease resistance.

Collectively, these results indicated that the intestinal microbiome might regulate the genes that were particularly involved in the steroid, steroid hormone, and BA biosynthesis to help the host resist *V. harveyi* infection through the microbe-intestine-immunity axis.

### Biosynthesis of unsaturated fatty acids

In the biosynthesis of the UFA pathway, we found the elongation of very-long-chain fatty acids protein 5 (*elovl5*) gene was down-expressed, while the acyl-CoA oxidase (*acox1*) gene and acyl-coenzyme A thioesterase 1/2/4 (*acot1_2_4*) gene was up-expressed in the *C. semilaevis* from resistant family, which promoted the biosynthesis of the UFAs (Fig. 6B). It was reported that appropriate levels of high UFA (HFUA) could significantly improve the disease resistance to vibriosis in grouper and parasites infections in large yellow croaker [67, 68], indicating lower infection in *C. semilaevis* from the resistant family. The *elovl5* gene was associated with *Alicyclobacillus* which was more abundant in the susceptible family (Fig. 6B), while this microbe could promote and/or aggravate inflammatory responses [51]. Accordingly, the lower abundance of *Alicyclobacillus* may reduce the proinflammatory cytokine level in the resistant family. Moreover, we observed that the *acox1* gene and *acot1_2_4* gene had significant associations with enriched abundances of *uc_Hyphomicrobiaceae* and *Propionibacterium*, respectively (Fig. 6B). The microbe *Propionibacterium* is considered as a candidate for probiotic due to the productivity of short-chain fatty acids [47, 48] and was useful for pathogen control in poultry rearing [48]. Thus, we boldly speculated that the intestinal microbes, especially the *Propionibacterium*, might play important roles in modulating the host UFA metabolism to resist vibriosis in *C. semilaevis*.

### Immune signaling transduction pathways

The KEGG analysis also revealed that the DEGs associated with the intestinal microbiome were enriched in three immune signaling transduction pathways, including Toll-like receptor signaling pathway, RIG-I-like receptor signaling pathway, and cytosolic DNA-sensing pathway (*p* < 0.05, Fig. 6C). These pathways could promote the biosynthesis of inflammatory cytokines such as TNF, IL6/12/1β, and IFNs, and thus strengthen the inflammation [69]. Excessive immune activation promotes the development of inflammation with perturbed physiological functions [70]. Therefore, the down-expressions of *rpc11*, *ifr3*, and *akt* genes in the resistant family indicated a lower inflammatory level, which might restrain excessive immune responses to maintain immunological homeostasis in *C. semilaevis*. Furthermore, the expression levels of DEGs enriched in immune signaling transduction pathways were correlated with the relative abundance of specific microbes (Fig. 6C). For example, *akt* and *rpc11* genes were associated with the resistant family-enriched *uc_Hyphomicrobiaceae* and *Phaeobacter*. While *Phaeobacter* was confirmed to be involved in immune system by regulating its *trxA* gene (Supplementary Figure 3 and Supplementary Table 3), which further suggested the microbes might mediate intestinal inflammation and immunological homeostasis through the microbe-intestine-immunity axis. Notably, the *irf3* gene was commonly involved in the Toll-like, RIG-I-like, and DNA-sensing pathways (Fig. 6C), thus may act as a central regulator in the interrelated immune signal-transduction schemes in reducing inflammation against vibriosis resistance in the resistant family.

Taken together, these results indicated that the host-microbiome interactions may mediate vibriosis resistance by regulating the immunological inflammations through controlling the lipid metabolism and immune signaling transduction pathways. All of these efforts are undoubtedly beneficial for higher production and better safety control for *C. semilaevis*. We also noticed that the regulative roles of most intestinal microbes we identified were not previously reported, probably due to limited knowledge in this field. In addition, numerous studies have shown the effect of the water microbiome on the intestinal microbiome of aquatic products [71, 72], though, in this study, we did not analyze the water microbiome, we also discovered *Pseudomonas*, a common member of the water microbiome [73], was dominant in the intestinal microbiome of *C. semilaevis* (Fig. 3A). Besides, this genus was associated with immunity-related DEGs (Fig. 5). Thus, further investigation is warranted to consider the potential effect of heterogeneous aquatic systems on the physiological characteristics and regulative mechanism of the intestinal microbiome in *C. semilaevis* against vibriosis.

### Combinatorial intestinal microbial and host functional markers for discriminating resistant family from susceptible family

**Fig. 7:**
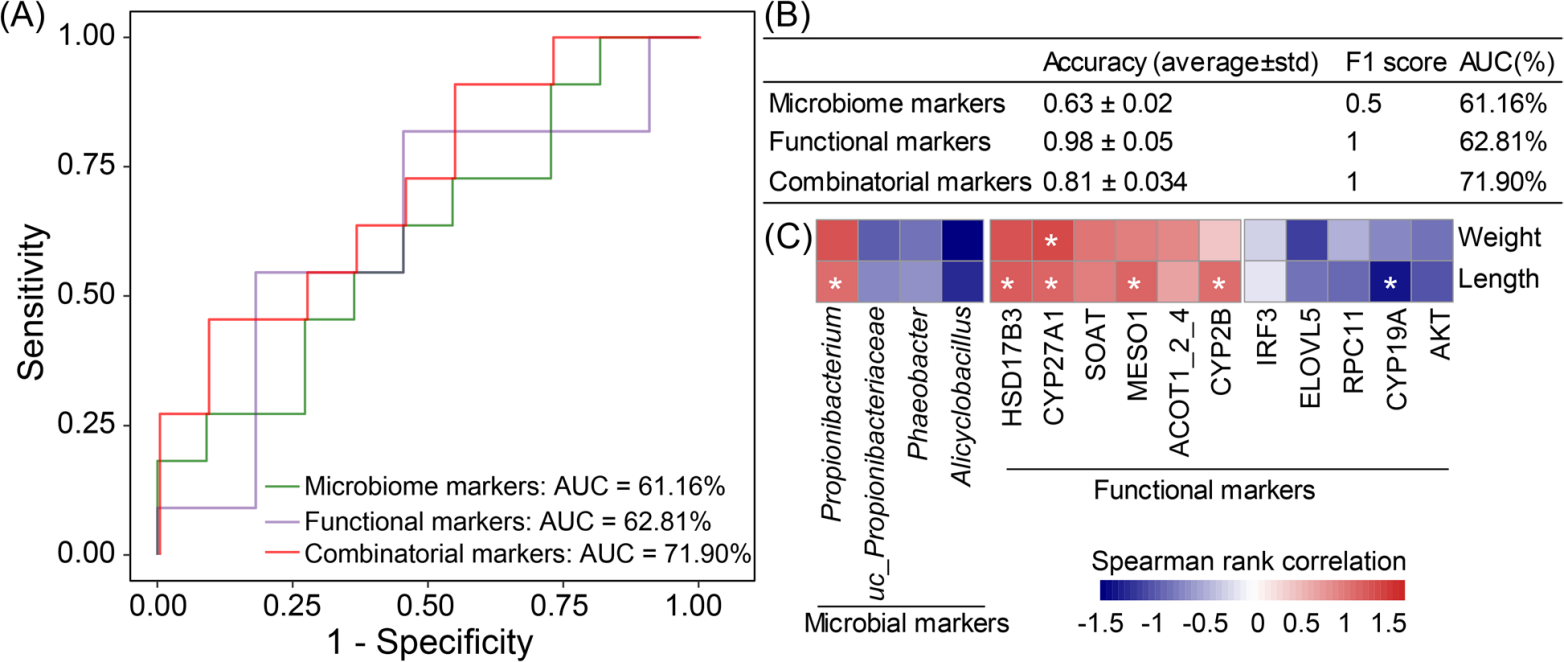
Multiple types of markers for discriminating the resistant family from the susceptible family in *C. semilaevis*. **A** Comparison of area under receiver operating characteristic curves (AUC), microbial markers (purple curve), functional markers (green curve), and combinatorial markers (red curve). **B** The AUC, F1 score, and accuracy of microbial markers, functional markers, and combinatorial markers. Four intestinal microbes: *Alicyclobacillus*, *uc_Propionibacteriaceae*, *Phaeobacter*, and *Propionibacterium* were selected as microbial markers due to their outstanding performance. The functional markers were the host DEGs (|log_2_(FoldChange)| > 1 and adjusted *p* < 0.05) which were significantly correlated with microbes (microbes: relative abundance ≥ 0.1% and coverage > 10% samples; correlation: |Spearman rank correlation| ≥ 0.5 and adjusted *p* < 0.05). These DEGs were mapped to 11 KO genes (*soat*, *meso1*, *hsd17b3*, *cyp27a1*, *acot1_2_4*, *cyp2b*, *cyp19a*, *irf3*, *rpc11*, *elovl5*, and *akt*), which were listed in **C**. **C** The correlations between multiple markers and growth characters (body weight and length). Only the Spearman rank correlations with *p* < 0.05 were considered as significant ones and indicated by “*”

Combinatorial markers could robustly discriminate the resistant family from the susceptible family. Here, we investigated the potential power of the intestinal microbes and host functional genes in discriminating the resistant family from the susceptible family. We found that four microbial markers: *Alicyclobacillus*, *uc_Propionibacteriaceae*, *Phaeobacter*, and *Propionibacterium* could accurately discriminate the resistant family from the susceptible family (area under curve (AUC): 61.16%, accuracy: 0.63 ± 0.02, and F1 score: 0.50; green curve; Fig. 7A, B) than that of other combination of microbial markers (Supplementary Figure 4). As for functional markers, eleven host genes (*soat*, *meso1*, *hsd17b3*, *cyp27a1*, *acot1_2_4*, *cyp2b*, *cyp19a*, *irf3*, *rpc11*, *elovl5*, *akt*; listed in Fig. 7C) showed outstanding performance (AUC: 62.81%, accuracy: 0.98 ± 0.05, and F1 score: 1; purple curve; Fig. 7A, B), out of other combinations of functional markers (Supplementary Figure 4). We then combined these microbial and functional markers, which enabled the highest prediction power in discriminating the resistant family from the susceptible family (AUC: 71.90%, accuracy: 0.81 ± 0.03, and F1 score: 0.95; red curve; Fig. 7A, B), yielding a more robust classification performance over that of the microbial and functional markers alone. These results suggested the host intestinal gene functional profiles have larger differences between the susceptible and resistant families, compared to the microbial community.

Interestingly, the microbial and functional markers were profoundly associated with the phenotypic characters, represented by body weight (BW) and length (BL) of *C. semilaevi*. Based on the combinatorial marker panel: 4 intestinal microbial markers (*Alicyclobacillus*, *uc_Propionibacteriaceae*, *Phaeobacter*, and *Propionibacterium*) and 11 host functional markers (*soat*, *meso1*, *hsd17b3*, *cyp27a1*, *acot1_2_4*, *cyp2b*, *cyp19a*, *irf3*, *rpc11*, *elovl5*, *akt*), we also sought into their association with phenotypic characters. The Spearman rank correlation analysis revealed one intestinal microbe (*Propionibacterium*: cor(relation) = 0.50, *p* = 0.04) and four genes (*hsd17b3*: cor = 0.55, *p* = 0.02; *cyp27a1*: cor = 0.54, *p* = 0.03; *meso1*: cor = 0.53, *p* = 0.03; and *cyp2b*: cor = 0.50, *p* = 0.04) were strongly positively correlated with the BL phenotype, while the *cyp19a* gene (cor = −0.49, *p* = 0.04) had a negative correlation (Fig. 7C). In addition, *cyp27a1* (cor = 0.49, *p* = 0.049) was also positively correlated with the BW phenotype (Fig. 7C). All these associated genes are involved in the cholesterol and BA biosynthesis pathways (Fig. 6A), indicating that cholesterol and BA metabolism were tightly correlated with the growth of *C. semilaevis*. Deng et al. have also confirmed dietary cholesterol supplementation promotes the growth in rainbow trout [74]. Additionally, the significant association of *Propionibacterium* with the growth characters highlighted that *Propionibacterium*, a candidate for probiotics and anti-pathogenic microbe [47, 48], might facilitate both the vibriosis resistance and growth of *C. semilaevis*. Collectively, these results further implied the important roles of the intestinal microbiome in disease resistance for *C. semilaevis*. Though previous works have reported that the intestinal microbes and metabolites could classify the types of fish [13, 75], those results depend on single types of omics data, and their robustness needs further verification. On the contrary, the features in the combinatorial marker panel are robust in classification, and the importance of these features is highly interpretable. Collectively, the combinatorial microbial and functional markers could be considered as potential features to facilitate the selective breeding of the vibriosis*-*resistant and fast-growing *C. semilaevis* germplasms in aquaculture.

## Conclusions

In this study, we have investigated the divergence of microbiomes and host-microbiome interactions underlying the vibriosis resistance in an economically important fish *C. semilaevis*. The *C. semilaevis* samples we have used in this study were the results over 10 years of selective breeding, resulting in vibriosis-resistant and susceptible families, which have formed an excellent model for investigating the interplay of host and intestinal microbiome in vibriosis resistance.

We found the host gene expressions, as well as the intestinal microbiome profiles, were distinct between the resistant and susceptible families in *C. semilaevis*. Additionally, our results indicated that the intestinal microbiome may shape the host metabolic and immune pathways underlying the vibriosis resistance in fish. The intestinal microbiome regulates the DEGs, which were up-expressed in steroid, steroid hormone, bile acid, and unsaturated fatty acid metabolism pathways; and down-expressed in the cytosolic DNA-sensing, Toll-like, and RIG-I-like receptor signaling pathways in the resistant family. Both of these up-expressions and down-expressions could help the resistant family of *C. semilaevis* to reduce the inflammation level. More importantly, the host-microbiome interactions in the lipid metabolism and immune signaling transduction pathways may in concert regulate the host immunological homeostasis and inflammatory levels and thus contribute to the improvement of vibriosis resistance. Furthermore, the combinatorial marker panel including intestinal microbial and host functional features could robustly discriminate the resistant and susceptible families, and these features were profoundly associated with the phenotypic characters, indicating that they could be applied to selective breeding for the vibriosis-resistant *C. semilaevis* germplasms in aquaculture.

Taken together, this study has demonstrated a better understanding of the intestinal microbiome and its functions against vibriosis for *C. semilaevis.* Our study, to the best of our knowledge, is one of the first reports using multi-omics to disentangle the host-microbiome interactions underpinning disease resistance in fish. Our study has provided new and important insights into the microbe-intestine-immunity axis for understanding the disease resistance in fish, and the discovered knowledge of the host-microbiome interactions could be applied for probiotics development and genetic modification to enhance the disease resistance and productivity in fish aquaculture.

## Supplementary Information

**Additional file 1: Supplementary Figure 1**. The alpha diversity of ARG Type in the intestinal microbiome of *C. semilaevis*. Except for Simpson, the diversity measured by ACE, Chao 1, good_coverage, observed species, Shannon, and was higher in the resistant family compared with susceptible family, but not significantly between two families. **Supplementary Figure 2**. The family-specific KEGG enrichment pathways of microbial genes. Circles and triangles represent the resistant family-specific and susceptible family-specific KEGG enrichment pathways, respectively. The color indicates the enrichment level (adjusted *p* < 0.05), the more enrichment of the pathway, the smaller the p value. The size of circles and triangles was dependent on the gene ratio. **Supplementary Figure 3**. The locations of *Phaeobacter* genes involved in lipid metabolism and immune pathways. Here we have used the assembled bins assigned to *Phaeobacter* for locating *Phaeobacter* genes. One bold arrow represents a KO gene, and its length indicates the length of the gene. The color of gene name represents the KO pathway of this gene involved. **Supplementary Figure 4**. Intestinal microbial, host functional, and combinatorial markers for discriminating the resistant family from the susceptible family in *C. semilaevis*. The accuracy, F1 score, and AUC were used for evaluating the performance of all microbes (yellow curve), top 4 important microbes (blue curve), top 3 important microbes (orange curve), 11 ko genes (the microbiome-associated DEGs significantly enriched pathways; green curve), top 5 important ko genes (pink curve) of 11 ko genes, top 5 important ko genes (wathet curve) of 11 ko genes, and the combination of top 4 important microbes and 11 ko genes (red curves). **Supplementary Table 1**. Detailed sample information of the *Cynoglossus semilaevis* samples collected from resistant and susceptible families. **Supplementary Table 2**. The detailed information of top 20 KEGG enrichment pathways (adjusted *p* < 0.05) for un- the down-expressed genes in the resistant family, respectively. **Supplementary Table 3**. The KEGG pathway of 8 differentially expressed genes between the resistant and susceptible families. **Supplementary Table 4**. The locations of *Phaeobacter* genes involved in lipid metabolism and immune pathways.

## Data Availability

Raw data for metagenomic sequencing and host RNA-seq for *C. semilaevis* project can be accessed at Genome Sequence Archive (GSA) database (https://ngdc.cncb.ac.cn/gsa/) with accession number PRJCA008512 and PRJCA010628, respectively.
