# Supplementary material for: Intestinal microbiome-mediated resistance against vibriosis for Cynoglossus semilaevis
Source: Microbiome. 2022 Sep 23;10:153. doi: 10.1186/s40168-022-01346-4 (PMC9503257; doi:10.1186/s40168-022-01346-4)
Supplement: Supplementary file 2 — Additional file 1: Supplementary Figure 1. The alpha diversity of ARG Type in the intestinal microbiome of C. semilaevis. Except for Simpson, the diversity measured by ACE, Chao 1, good_coverage, observed species, Shannon, and was higher in the resistant family compared with susceptible family, but not significantly between two families. Supplementary Figure 2. The family-specific KEGG enrichment pathways of microbial genes. Circles and triangles represent the resistant family-specific and susceptible family-specific KEGG enrichment pathways, respectively. The color indicates the enrichment level (adjusted p < 0.05), the more enrichment of the pathway, the smaller the p value. The size of circles and triangles was dependent on the gene ratio. Supplementary Figure 3. The locations of Phaeobacter genes involved in lipid metabolism and immune pathways. Here we have used the assembled bins assigned to Phaeobacter for locating Phaeobacter genes. One bold arrow represents a KO gene, and its length indicates the length of the gene. The color of gene name represents the KO pathway of this gene involved. Supplementary Figure 4. Intestinal microbial, host functional, and combinatorial markers for discriminating the resistant family from the susceptible family in C. semilaevis. The accuracy, F1 score, and AUC were used for evaluating the performance of all microbes (yellow curve), top 4 important microbes (blue curve), top 3 important microbes (orange curve), 11 ko genes (the microbiome-associated DEGs significantly enriched pathways; green curve), top 5 important ko genes (pink curve) of 11 ko genes, top 5 important ko genes (wathet curve) of 11 ko genes, and the combination of top 4 important microbes and 11 ko genes (red curves). Supplementary Table 1. Detailed sample information of the Cynoglossus semilaevis samples collected from resistant and susceptible families. Supplementary Table 2. The detailed information of top 20 KEGG enrichment pathways (adjusted p < 0.05) for u [file 40168_2022_1346_MOESM1_ESM.docx]

**Supplementary materials**

**Intestinal microbiome-mediated resistance against** **vibriosis for *Cynoglossus* *semilaevis***

Qian Zhou^1,$^, Xue Zhu^2,$^, Yangzhen Li^1^, Pengshuo Yang^2^, Shengpeng Wang^3^, Kang Ning^2,*^, Songlin Chen^1,*^

^1^Yellow Sea Fisheries Research Institute, Chinese Academy of Fishery Sciences / Key Laboratory for Sustainable Development of Marine Fisheries, Ministry of Agriculture; Shandong Key Laboratory for Marine Fishery Biotechnology and Genetic Breeding; Laboratory for Marine Fisheries Science and Food Production Processes, Pilot National Laboratory for Marine Science and Technology (Qingdao), Qingdao 266071, Shandong, China

^2^Key Laboratory of Molecular Biophysics of the Ministry of Education, Hubei Key Laboratory of Bioinformatics and Molecular-imaging, Center of AI Biology, Department of Bioinformatics and Systems Biology, College of Life Science and Technology, Huazhong University of Science and Technology, Wuhan 430074, Hubei, China

^3^Dezhou Key Laboratory for Applied Bile Acid Research, Shandong Longchang Animal Health Product Co., Ltd, Qihe, Shandong Lachance Co., Ltd, Jinan 251100, Shandong, China

^$^These authors contributed equally to this work

^*^Corresponding authors: Kang Ning (Email: ningkang@hust.edu.cn) and Songlin Chen (Email: chensl@ysfri.ac.cn)

**Supplementary Figures**


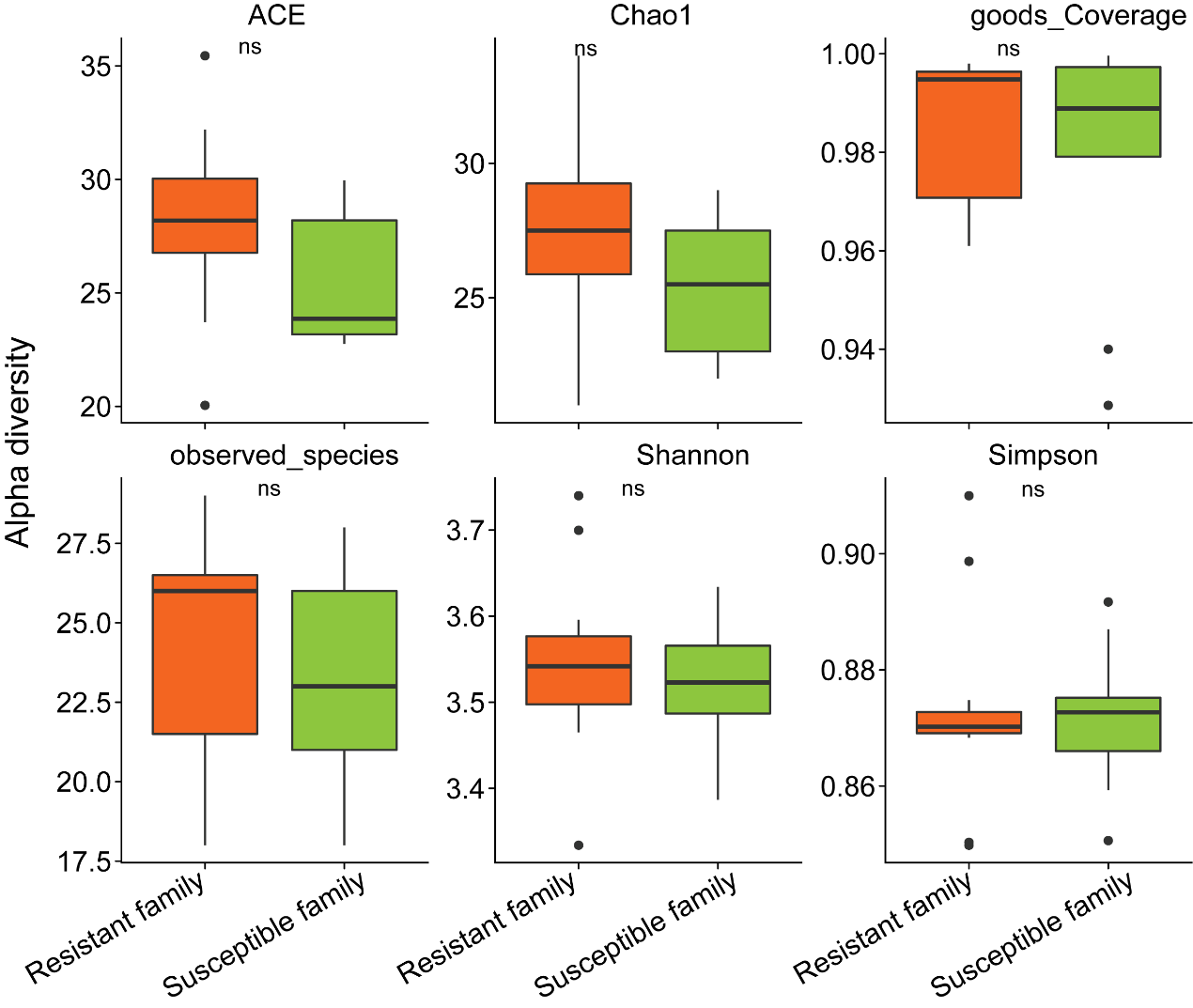


**Supplementary Figure 1. The alpha diversity of ARG Type in the intestinal microbiome of *C. semilaevis*.** Except for Simpson, the diversity measured by ACE, Chao 1, good_coverage, observed species, Shannon, and was higher in the resistant family compared with susceptible family, but not significantly between two families.

**
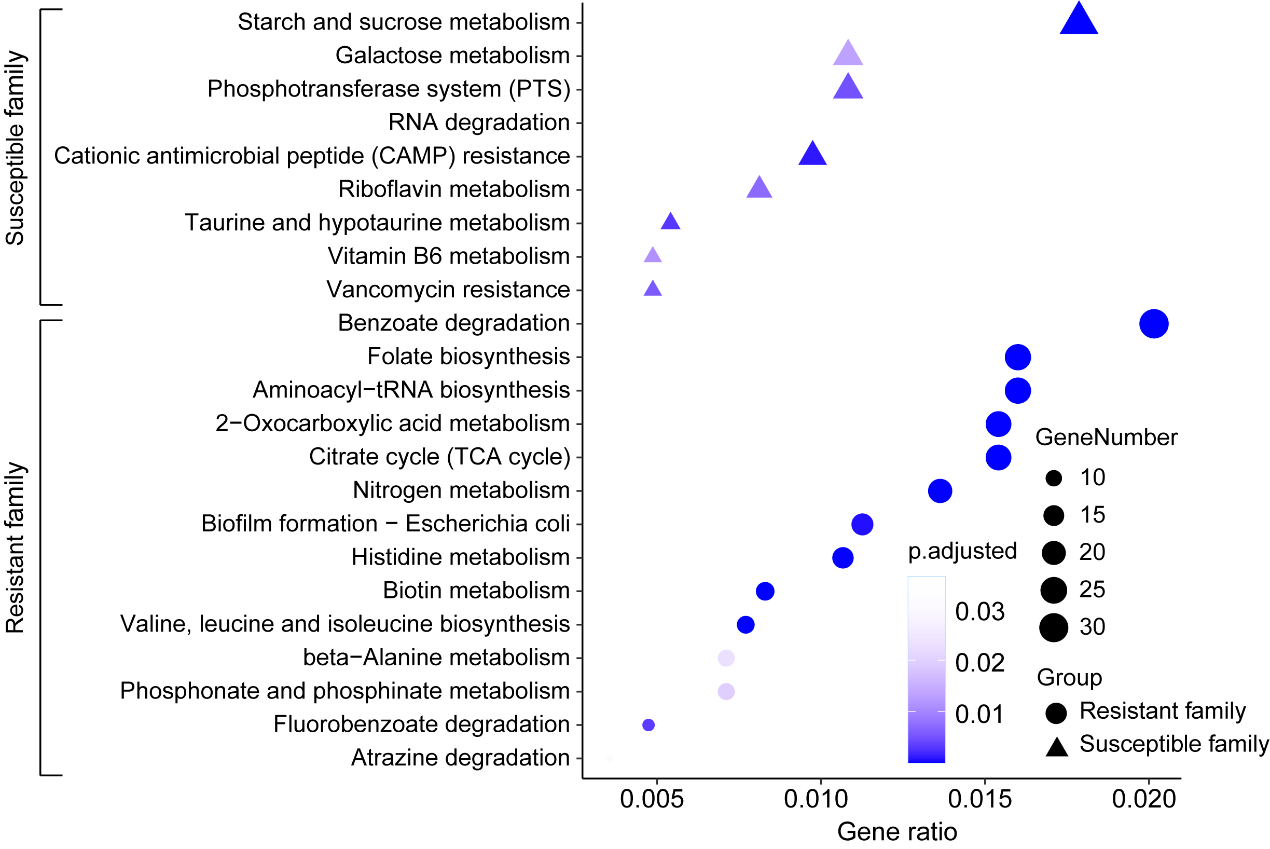
**

**Supplementary Figure 2. The family-specific KEGG enrichment pathways of microbial genes.** Circles and triangles represent the resistant family-specific and susceptible family-specific KEGG enrichment pathways, respectively. The color indicates the enrichment level (adjusted p < 0.05), the more enrichment of the pathway, the smaller the p value. The size of circles and triangles was dependent on the gene ratio.


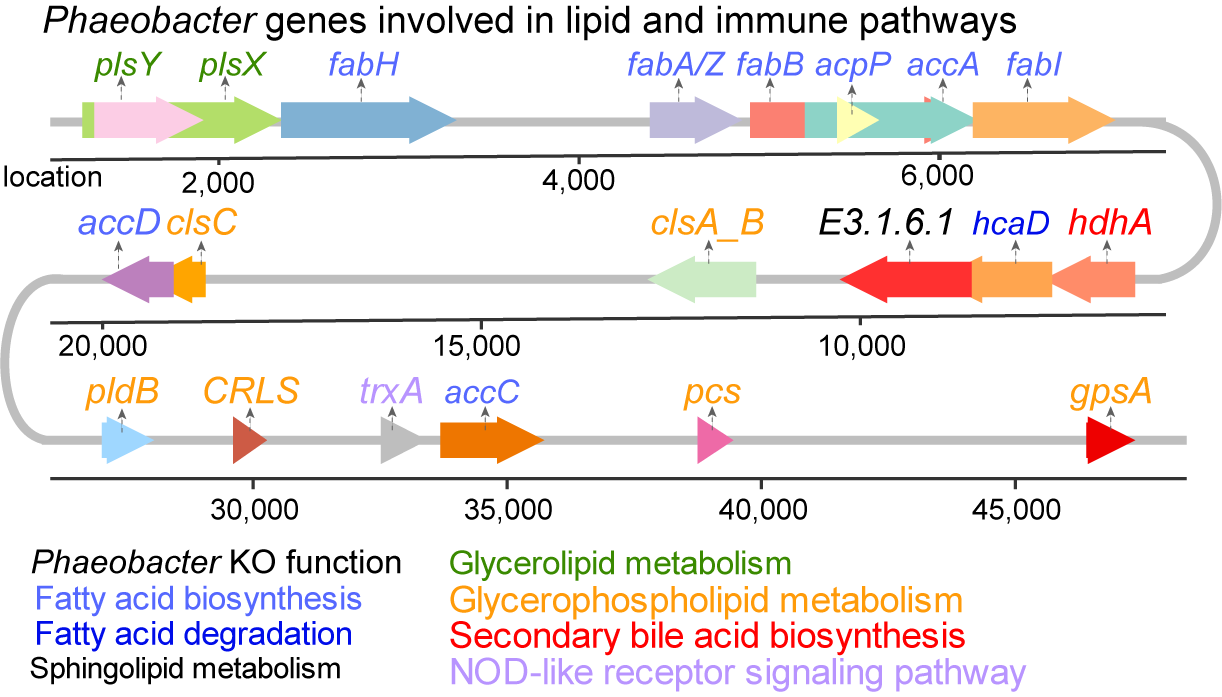


**Supplementary Figure 3. The locations of *Phaeobacter* genes involved in lipid metabolism and immune pathways.** Here we have used the assembled bins assigned to *Phaeobacter* for locating *Phaeobacter* genes. One bold arrow represents a KO gene, and its length indicates the length of the gene. The color of gene name represents the KO pathway of this gene involved.


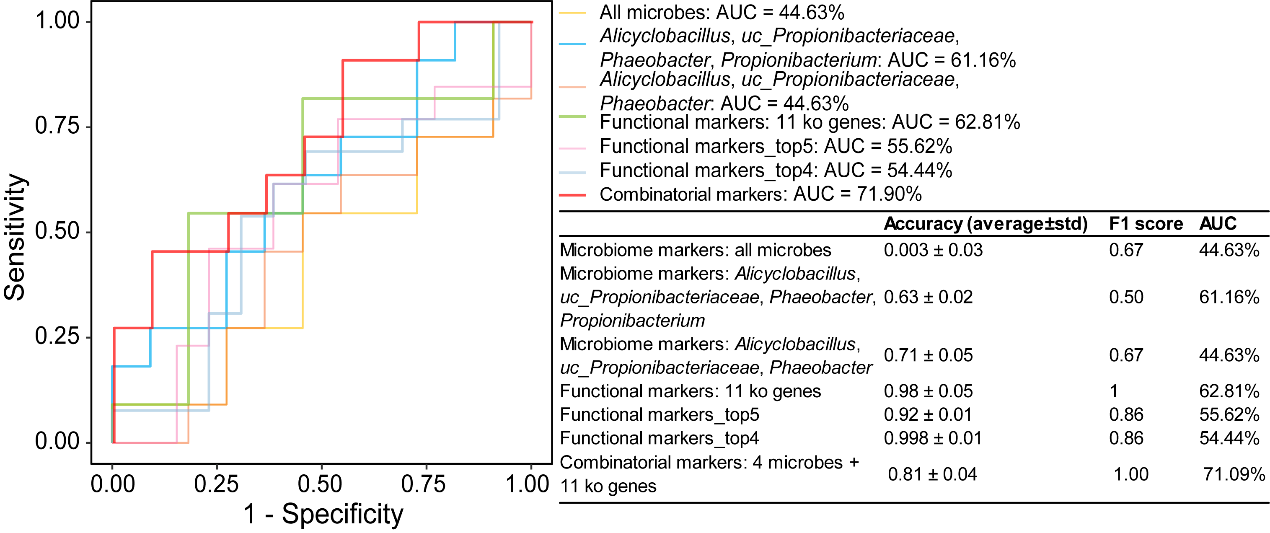


**Supplementary Figure 4. Intestinal microbial, host functional, and combinatorial markers for discriminating the resistant family from the susceptible family in *C. semilaevis*.** The accuracy, F1 score, and AUC were used for evaluating the performance of all microbes (yellow curve), top 4 important microbes (blue curve), top 3 important microbes (orange curve), 11 ko genes (the microbiome-associated DEGs significantly enriched pathways; green curve), top 5 important ko genes (pink curve) of 11 ko genes, top 5 important ko genes (wathet curve) of 11 ko genes, and the combination of top 4 important microbes and 11 ko genes (red curves).

**Supplementary Tables**

**Supplementary Table 1. Detailed sample information of the *Cynoglossus semilaevis* samples collected from resistant and susceptible families.**

| **SampleID** | **Body weight (BW/g)** | **Body length (BL/cm)** | **Survival rate** | **Group** |
| --- | --- | --- | --- | --- |
| CS126 | 23.4 | 16.3 | 97.67% | Resistant family |
| CS146 | 22.8 | 16.5 |  |  |
| CS156 | 24.2 | 17.8 |  |  |
| CS186 | 19.5 | 16 |  |  |
| CS196 | 23.6 | 17.1 |  |  |
| CS1106 | 22.8 | 16.9 |  |  |
| CS316 | 15.5 | 14.3 | 91.67% |  |
| CS3106 | 23.3 | 15.7 |  |  |
| CS216 | 21.7 | 17.0 | 14.10% | Susceptible family |
| CS226 | 22.0 | 14.6 |  |  |
| CS236 | 16.8 | 12.5 |  |  |
| CS246 | 19.2 | 13.5 |  |  |
| CS256 | 22.5 | 17.7 |  |  |
| CS266 | 19.5 | 16 |  |  |
| CS286 | 19.3 | 16.1 |  |  |
| CS296 | 23.6 | 17.5 |  |  |
| CS2106 | 20.4 | 16.8 |  |  |

**Supplementary Table 2. The detailed information of top 20 KEGG enrichment pathways (adjusted p < 0.05) for un- the down-expressed genes in the resistant family, respectively.**

|  | KEGG pathway | Gene_num | p value | Pathway ID | Gene ID | K ID (correspoding to the rank of genes) |
| --- | --- | --- | --- | --- | --- | --- |
| Top 20 KEGG pathways enrichment of the up-expressed genes | Steroid biosynthesis | 3 | 0.001 | ko00100 | Cse_R000680;Cse_R019276;Cse_R021919 | K00637+K00637+K07750 |
|  | Intestinal immune network for IgA production | 3 | 0.003 | ko04672 | Cse_R001773;Cse_R002032;Cse_R007545 | K06752+K06752+K04189 |
|  | Cholesterol metabolism | 4 | 0.004 | ko04979 | Cse_R000680;Cse_R000348;Cse_R019276;Cse_R021396 | K00637+K08760+K00637+K00488 |
|  | Glutamatergic synapse | 6 | 0.004 | ko04724 | Cse_R003359;Cse_R005392;Cse_R009499;Cse_R009525;Cse_R013906;Cse_R018755 | K04605+K02677+K05199+K05199+K04604+K05612 |
|  | Hematopoietic cell lineage | 4 | 0.005 | ko04640 | Cse_R001773;Cse_R002032;Cse_R004678;Cse_R020572 | K06752+K06752+K06467+K05072 |
|  | PPAR signaling pathway | 4 | 0.008 | ko03320 | Cse_R005825;Cse_R015228;Cse_R015305;Cse_R021396 | K08524+K00232+K06086+K00488 |
|  | Asthma | 2 | 0.010 | ko05310 | Cse_R001773;Cse_R002032 | K06752+K06752 |
|  | Retinol metabolism | 3 | 0.011 | ko00830 | Cse_R008454;Cse_R017588;Cse_R020987 | K11150+K07412+K09516 |
|  | Th17 cell differentiation | 4 | 0.015 | ko04659 | Cse_R001773;Cse_R002032;Cse_R005825;Cse_R010077 | K06752+K06752+K08524+K05060 |
|  | Graft-versus-host disease | 2 | 0.015 | ko05332 | Cse_R001773;Cse_R002032 | K06752+K06752 |
|  | Neuroactive ligand-receptor interaction | 8 | 0.018 | ko04080 | Cse_R002710;Cse_R003359;Cse_R009499;Cse_R009525;Cse_R009535;Cse_R009604;Cse_R012334;Cse_R013906 | K04228+K04605+K05199+K05199+K04142+K04136+K04230+K04604 |
|  | Biosynthesis of unsaturated fatty acids | 2 | 0.022 | ko01040 | Cse_R010665;Cse_R015228 | K01068+K00232 |
|  | Allograft rejection | 2 | 0.023 | ko05330 | Cse_R001773;Cse_R002032 | K06752+K06752 |
|  | Autoimmune thyroid disease | 2 | 0.030 | ko05320 | Cse_R001773;Cse_R002032 | K06752+K06752 |
|  | Phospholipase D signaling pathway | 5 | 0.034 | ko04072 | Cse_R002710;Cse_R003359;Cse_R005392;Cse_R013906;Cse_R021117 | K04228+K04605+K02677+K04604+K01528 |
|  | Long-term depression | 3 | 0.034 | ko04730 | Cse_R005392;Cse_R009499;Cse_R009525 | K02677+K05199+K05199 |
|  | Type I diabetes mellitus | 2 | 0.036 | ko04940 | Cse_R001773;Cse_R002032 | K06752+K06752 |
|  | Choline metabolism in cancer | 4 | 0.050 | ko05231 | Cse_R003988;Cse_R004247;Cse_R005392;Cse_R019183 | K14387+K14387+K02677+K08202 |
|  | Retrograde endocannabinoid signaling | 4 | 0.059 | ko04723 | Cse_R005392;Cse_R009499;Cse_R009525;Cse_R013906 | K02677+K05199+K05199+K04604 |
|  | Steroid hormone biosynthesis | 2 | 0.062 | ko00140 | Cse_R009333;Cse_R017588 | K10207+K07412 |
| Top 21 KEGG pathways enrichment of the down-expressed genes | Hepatitis C | 7 | 0.000 | ko05160 | Cse_R003826;Cse_R004106;Cse_R009320;Cse_R012801;Cse_R014797;Cse_R015377;Cse_R021377 | K14754+K04456+K04382+K09447+K05411+K14217+K11220 |
|  | RIG-I-like receptor signaling pathway | 4 | 0.001 | ko04622 | Cse_R007565;Cse_R008416;Cse_R012801;Cse_R014797 | K12652+K12649+K09447+K05411 |
|  | Measles | 5 | 0.001 | ko05162 | Cse_R003826;Cse_R004106;Cse_R012801;Cse_R014797;Cse_R021377 | K14754+K04456+K09447+K05411+K11220 |
|  | Cytosolic DNA-sensing pathway | 3 | 0.002 | ko04623 | Cse_R006552;Cse_R012801;Cse_R014797 | K03019+K09447+K05411 |
|  | Toll-like receptor signaling pathway | 4 | 0.002 | ko04620 | Cse_R004106;Cse_R012801;Cse_R014797;Cse_R021377 | K04456+K09447+K05411+K11220 |
|  | Glutathione metabolism | 3 | 0.003 | ko00480 | Cse_R001258;Cse_R009248;Cse_R012963 | K01581+K10807+K11140 |
|  | Influenza A | 5 | 0.006 | ko05164 | Cse_R003826;Cse_R004106;Cse_R012801;Cse_R014797;Cse_R021377 | K14754+K04456+K09447+K05411+K11220 |
|  | Amino sugar and nucleotide sugar metabolism | 3 | 0.008 | ko00520 | Cse_R003085;Cse_R019999;Cse_R021650 | K01183+K01784+K05305 |
|  | Mucin type O-glycan biosynthesis | 2 | 0.011 | ko00512 | Cse_R005534;Cse_R012983 | K09663+K09662 |
|  | Adrenergic signaling in cardiomyocytes | 5 | 0.014 | ko04261 | Cse_R002648;Cse_R004106;Cse_R009026;Cse_R009320;Cse_R015561 | K05850+K04456+K01539+K04382+K04867 |
|  | mRNA surveillance pathway | 3 | 0.019 | ko03015 | Cse_R003536;Cse_R009320;Cse_R014439 | K14326+K04382+K06965 |
|  | Hepatitis B | 4 | 0.030 | ko05161 | Cse_R004106;Cse_R012801;Cse_R014797;Cse_R021377 | K04456+K09447+K05411+K11220 |
|  | Epstein-Barr virus infection | 4 | 0.036 | ko05169 | Cse_R004106;Cse_R012801;Cse_R014797;Cse_R021377 | K04456+K09447+K05411+K11220 |
|  | Carbohydrate digestion and absorption | 2 | 0.037 | ko04973 | Cse_R004106;Cse_R009026 | K04456+K01539 |
|  | Kaposi sarcoma-associated herpesvirus infection | 4 | 0.039 | ko05167 | Cse_R004106;Cse_R012801;Cse_R014797;Cse_R021377 | K04456+K09447+K05411+K11220 |
|  | Mineral absorption | 2 | 0.044 | ko04978 | Cse_R002648;Cse_R009026 | K05850+K01539 |
|  | Metabolic pathways | 15 | 0.051 | ko01100 | Cse_R016502;Cse_R001258;Cse_R003085;Cse_R004063;Cse_R005534;Cse_R006301;Cse_R009248;Cse_R012562;Cse_R012963;Cse_R012983;Cse_R018040;Cse_R019999;Cse_R021650;Cse_R020833;Cse_R020948 | K00851+K01581+K01183+K10244+K09663+K01638+K10807+K07434+K11140+K09662+K00923+K01784+K05305+K01832+K07970 |
|  | Human papillomavirus infection | 6 | 0.059 | ko05165 | Cse_R003826;Cse_R004106;Cse_R009320;Cse_R014797;Cse_R014951;Cse_R021377 | K14754+K04456+K04382+K05411+K04659+K11220 |
|  | Non-homologous end-joining | 1 | 0.066 | ko03450 | Cse_R005285 | K03513 |
|  | cGMP - PKG signaling pathway | 4 | 0.075 | ko04022 | Cse_R002648;Cse_R004106;Cse_R009026;Cse_R020212 | K05850+K04456+K01539+K04952 |

**Supplementary Table 3. The KEGG pathway of 8 differentially expressed genes between the resistant and susceptible families.**

| KID | geneName | Level1 | Level2 | Level3 | Level4 | Resitiant family (TPM) | Susceptible family (TPM) | p |
| --- | --- | --- | --- | --- | --- | --- | --- | --- |
| K02314 | *dnaB* | Cellular Processes | Cell growth and death | Cell cycle - Caulobacter | replicative DNA helicase [EC:3.6.4.12] | 82.7363 | 207.8228 | 0.0196 |
| K07793 | *tctA* | Environmental Information Processing | Signal transduction | Two-component system | putative tricarboxylic transport membrane protein | 4.0266 | 34.3853 | 0.0314 |
| K02939 | *RP-L9, MRPL9, rplI* | Genetic Information Processing | Translation | Ribosome | large subunit ribosomal protein L9 | 80.7559 | 242.6001 | 0.0421 |
| K01839 | *deoB* | Metabolism | Global and overview maps | Metabolic pathways | phosphopentomutase [EC:5.4.2.7] | 1.0326 | 25.4113 | 0.0411 |
| K01556 | *kynU* | Metabolism | Global and overview maps | Metabolic pathways | kynureninase [EC:3.7.1.3] | 0.6917 | 26.2805 | 0.0306 |
| K21620 | *sorbD* | Metabolism | Global and overview maps | Metabolic pathways | galactitol 2-dehydrogenase [EC:1.1.1.16] | 1.0566 | 17.5740 | 0.0488 |
| K03596 | *lepA* | Human Diseases | Infectious disease: bacterial | Legionellosis | GTP-binding protein LepA | 100.4222 | 24.8250 | 0.0194 |
| K03743 | *pncC* | Metabolism | Metabolism of cofactors and vitamins | Nicotinate and nicotinamide metabolism | nicotinamide-nucleotide amidase [EC:3.5.1.42] | 70.4423 | 14.0017 | 0.0449 |

**Supplementary Table 4. The locations of *Phaeobacter* genes involved in lipid metabolism and immune pathways.**

| geneID | KO genes | Start | End | Level1 | Level2 | Level3 | Level4 |
| --- | --- | --- | --- | --- | --- | --- | --- |
| NODE_92_length_10390_cov_5.816057_6 | *accA* | 5,249 | 6,211 | Metabolism | Lipid metabolism | Fatty acid biosynthesis | acetyl-CoA carboxylase carboxyl transferase subunit alpha |
| NODE_28_length_49742_cov_6.018021_32 | *accC* | 33,684 | 35,729 | Metabolism | Lipid metabolism | Fatty acid biosynthesis | acetyl-CoA carboxylase, biotin carboxylase subunit |
| NODE_22_length_58612_cov_6.619458_23 | *accD* | 22,678 | 23,625 | Metabolism | Lipid metabolism | Fatty acid biosynthesis | acetyl-CoA carboxylase carboxyl transferase subunit beta |
| NODE_64_length_21264_cov_5.130457_6 | *acpP* | 5,428 | 5,661 | Metabolism | Lipid metabolism | Fatty acid biosynthesis | acyl carrier protein |
| NODE_73_length_17399_cov_6.112574_5 | *fabH* | 2,342 | 3,316 | Metabolism | Lipid metabolism | Fatty acid biosynthesis | 3-oxoacyl- |
| NODE_98_length_8388_cov_6.176754_9 | *fabB* | 4,945 | 6,174 | Metabolism | Lipid metabolism | Fatty acid biosynthesis | 3-oxoacyl- |
| NODE_98_length_8388_cov_6.176754_8 | *fabA/Z* | 4,389 | 4,901 | Metabolism | Lipid metabolism | Fatty acid biosynthesis | 3-hydroxyacyl- |
| NODE_98_length_8388_cov_6.176754_10 | *fabI* | 6,182 | 6,973 | Metabolism | Lipid metabolism | Fatty acid biosynthesis | enoyl- |
| NODE_66_length_20530_cov_5.940155_9 | *hcaD* | 9,991 | 11,202 | Metabolism | Lipid metabolism | Fatty acid degradation | 3-phenylpropionate/trans-cinnamate dioxygenase ferredoxin reductase component |
| NODE_31_length_42890_cov_5.702310_27 | *hdhA* | 22,256 | 23,059 | Metabolism | Lipid metabolism | Secondary bile acid biosynthesis | 7-alpha-hydroxysteroid dehydrogenase |
| NODE_73_length_17399_cov_6.112574_4 | *plsX* | 1,239 | 2,345 | Metabolism | Lipid metabolism | Glycerolipid metabolism | phosphate acyltransferase |
| NODE_68_length_19732_cov_6.328364_2 | *plsY* | 1,305 | 1,910 | Metabolism | Lipid metabolism | Glycerolipid metabolism | acyl phosphate:glycerol-3-phosphate acyltransferase |
| NODE_15_length_72408_cov_6.998189_50 | *gpsA* | 46,395 | 47,354 | Metabolism | Lipid metabolism | Glycerophospholipid metabolism | glycerol-3-phosphate dehydrogenase (NAD(P)+) |
| NODE_20_length_59053_cov_6.181498_32 | *pldB* | 27,025 | 28,050 | Metabolism | Lipid metabolism | Glycerophospholipid metabolism | lysophospholipase |
| NODE_27_length_50611_cov_6.802964_36 | *pcs* | 38,745 | 39,449 | Metabolism | Lipid metabolism | Glycerophospholipid metabolism | phosphatidylcholine synthase |
| NODE_37_length_35622_cov_6.591993_14 | *clsA_B* | 14,990 | 16,423 | Metabolism | Lipid metabolism | Glycerophospholipid metabolism | cardiolipin synthase A/B |
| NODE_60_length_21772_cov_5.470201_12 | *clsC* | 11,084 | 12,631 | Metabolism | Lipid metabolism | Glycerophospholipid metabolism | cardiolipin synthase C |
| NODE_12_length_76999_cov_6.671433_29 | *CRLS* | 29,608 | 30,273 | Metabolism | Lipid metabolism | Glycerophospholipid metabolism | cardiolipin synthase (CMP-forming) |
| NODE_32_length_40737_cov_6.745475_14 | *E3.1.6.1* | 12,148 | 13,887 | Metabolism | Lipid metabolism | Sphingolipid metabolism | arylsulfatase |
| NODE_4_length_106514_cov_6.290068_33 | *trxA* | 32,516 | 33,427 | Organismal Systems | Immune system | NOD-like receptor signaling pathway | thioredoxin 1 |
